# Analysis of risk factors for severe acute poisoning in children

**DOI:** 10.3389/fped.2026.1903927

**Published:** 2026-08-07

**Authors:** Jing Li, Wei Kai Wang

**Affiliations:** 1 The First Clinical Medical College, Gansu University of Chinese Medicine, Lanzhou, China; 2 Tianshui First People’s Hospital, Tianshui, China

**Keywords:** acute poisoning, children, poisoning severity score, risk factors, severe poisoning

## Abstract

**Objective:**

To identify independent risk factors for the progression of acute poisoning to severe cases in children, and to provide a basis for early clinical identification and intervention.

**Methods:**

A retrospective study was conducted to collect the clinical data of hospitalized children with acute poisoning at Tianshui First People's Hospital from January 1, 2018 to December 31, 2025. According to the Poisoning Severity Score (PSS), patients were divided into the severe group (≥3 points) and the non-severe group (<3 points). Variables that were statistically significant in univariate analysis were included in binary logistic regression analysis to identify independent risk factors for severe poisoning.

**Results:**

A total of 726 children with acute poisoning were enrolled, including 79 (10.9%) severe cases and 647 (89.1%) non-severe cases**.** Two deaths occurred, with a case fatality rate of 0.3%. The difference across age groups was statistically significant (*χ*^2^ = 10.451, *P* = 0.015), with adolescents accounting for the highest proportion of severe cases (48.1%, 38/79). Multivariate logistic regression analysis revealed that gastric lavage was significantly associated with lower odds of severe poisoning (*OR* = 0.355, 95% *CI*: 0.157–0.800, *P* = 0.013); antidote use was associated with an increased risk of severe poisoning (*OR* = 3.156, 95% *CI*: 1.933–5.152, *P* < 0.001), while left-behind children (*OR* = 3.013, 95% *CI*: 1.558–5.828, *P* = 0.001) and self-poisoning (*OR* = 2.113, 95% *CI*: 1.283–3.481, *P* = 0.003) were independent risk factors**.** The ROC curve showed an area under the curve (AUC) of 0.710 (95% CI: 0.645–0.775).

**Conclusions:**

Gastric lavage associated with a lower risk of severe poisoning in this pediatric cohort. When strictly indicated, it may remain a procedure of clinical value. Left-behind children and those with self-poisoning are high-risk populations requiring enhanced family supervision, early recognition, and psychological intervention. The association between antidote use and increased risk of severe poisoning likely reflects confounding by indication, and should not delay necessary antidotal therapy.

## 1 Introduction

Acute poisoning in children is one of the common clinical emergencies in pediatrics, characterized by sudden onset and rapid progression. Although many poisons lack specific antidotes, most cases can be appropriately managed with supportive care in a hospital setting, and most patients have good outcomes with no long-term morbidity. However, severe cases are often accompanied by multiple organ dysfunction and may even be life-threatening (1, 2). According to statistics, poisoning and trauma rank fifth among the causes of disease death in China (3). In children aged 1–4 years, poisoning is the leading cause of accidental death, with a mortality rate of 4.38% (4). Acute poisoning has seriously endangered the physical and mental health of children and caused a heavy burden on families and society. Early identification of risk factors for severe poisoning and timely intervention are particularly important for improving the prognosis of the disease and reducing the burden.

Recent studies have shown that the characteristics of acute poisoning in children are different in different regions, and the characteristics of acute poisoning are also different in the same region over different periods (5, 6). At present, limited research has been conducted on acute poisoning in this region, mostly focusing on the epidemiology and clinical characteristics of acute poisoning in children, with few studies on the risk factors for severe poisoning. This study aims to identify the independent risk factors for the progression of acute poisoning to severe cases in children through a retrospective analysis of clinical data of children with acute poisoning in our hospital, and to provide a basis for early clinical identification and intervention. To our knowledge, this study represents the first systematic analysis of risk factors for severe acute poisoning in children in the Tianshui region.

## 2 Subjects and methods

### 2.1 Study population

This retrospective study included children hospitalized for acute poisoning at Tianshui First People's Hospital between January 1, 2018 and December 31, 2025. The inclusion criteria were as follows: (1) diagnosis of acute poisoning according to the Chinese Expert Consensus on Diagnosis and Treatment of Acute Poisoning (2016 edition) (7); (2) a clear history of acute poisoning, defined as acute pathological changes caused by a single large-dose exposure or multiple exposures within 24 h to one or more toxic substances (7, 8); (3) children aged 1 month to 14 years inclusive. The exclusion criteria were: (1) chronic poisoning, defined as toxic accumulation in the body due to long-term exposure (8); (2) incomplete clinical data, defined as missing more than 20% of key clinical information (including but not limited to demographic data, time from exposure to hospital arrival, and poison type); (3) poisoning complicated by underlying diseases that may interfere with system involvement assessment, such as encephalitis, meningitis, other infectious diseases, status epilepticus, adrenal insufficiency, and hyperammonemia. For children with intellectual disability or developmental delay, those with a clear history of toxic substance ingestion or laboratory-confirmed poisoning were included; otherwise, they were excluded. The study protocol was approved by the Ethics Committee of Tianshui First People's Hospital (approval No. 2025-051).

### 2.2 Data collection and grouping

Data were extracted from the hospital's electronic medical record system. The collected variables included general information (age, sex), region of residence (urban or rural), type of poison, route and cause of poisoning, time from poison exposure to hospital arrival, clinical manifestations (involved systems), clinical interventions (gastric lavage, fluid infusion, antidote administration, and ventilator use), outcome, family factors (whether the child was left-behind or an only child), and whether pre-hospital first aid was provided by family members.

Poison types were reclassified into four categories: (1) Pesticides (organophosphates, rodenticides, herbicides, and insecticides); (2) Antidepressants; (3) Other drugs (sedatives, antibiotics, antihypertensives, etc.); (4) Others (chemical agents, animal bites, carbon monoxide, alcohol, poisonous mushrooms, kerosene, chlorine, Datura stramonium, etc.). Age groups were defined as: infants and toddlers (1 month to 3 years), preschool age (4–6 years), school age (7–9 years), and adolescence (10–14 years).

Time from poison exposure to hospital arrival was categorized into four groups: ≤1 h, 1–4 h, 4–6 h, and >6 h, based on the recommended time windows for gastric lavage (7).

Poisoning severity was graded according to the Poisoning Severity Score (PSS) (7, 9), which comprises five levels: asymptomatic (0 points, no signs or symptoms); mild (1 point, transient self-limiting symptoms or signs); moderate (2 points, pronounced persistent symptoms or signs with organ dysfunction); severe (3 points, severe life-threatening symptoms or signs with severe organ dysfunction); and death (4 points).

PSS was assessed at admission prior to any gastric lavage or antidote administration and at 24 h post-admission (after these interventions had been performed), with additional assessments performed as clinically indicated based on the patient's condition. The maximum PSS value within the first 24 h was used for group allocation (10). Patients were divided into the severe group (PSS ≥3) and the non-severe group (PSS <3).

### 2.3 Statistical analysis

Statistical analyses were performed using SPSS version 27.0. Categorical variables were presented as frequencies and percentages, and differences between groups were analyzed using the chi-square test. Variables with a *P* value < 0.05 in univariate analysis were entered into a binary logistic regression model (forward stepwise method), with entry criteria set at *P* < 0.05 and removal criteria at *P* > 0.10, based on the Akaike Information Criterion (AIC) to determine the final model. Odds ratios (*OR*) and 95% confidence intervals (*CI*) were calculated to identify independent risk factors for severe poisoning. A *P* value < 0.05 was considered statistically significant. Model calibration was assessed using the Hosmer-Lemeshow test, and predictive performance was evaluated by plotting the receiver operating characteristic (ROC) curve and calculating the area under the curve (AUC). Collinearity was examined using the variance inflation factor (VIF). Pairwise comparisons for age groups were performed with Bonferroni correction.

## 3 Results

### 3.1 Overall distribution and baseline characteristics of severe and non-severe poisoning

A total of 726 children with acute poisoning were included. Overall, 79 cases (10.9%) were in the severe group and 647 cases (89.1%) in the non-severe group, Two deaths occurred, with a case fatality rate of 0.3%. There were 354 boys (48.8%) and 372 girls (51.2%). Rural children accounted for 590 cases (81.3%) and urban children for 136 cases (18.7%). Regarding age distribution, the highest proportion was in adolescents (276 cases, 38.0%), followed by infants and toddlers (227 cases, 31.3%), preschool-aged children (149 cases, 20.5%), and school-aged children (74 cases, 10.2%). Adolescents were the most affected age group.

In the severe group, adolescents accounted for the highest proportion (48.1%, 38/79), self-poisoning accounted for 48.1% (38/79), and accidental poisoning for 51.9% (41/79). The most common poison type was pesticides (33 cases, 41.8%), followed by antidepressants (11 cases, 13.9%). Neurological involvement was the most frequent (96.2%). The rates of ventilator use and antidote use in the severe group were 11.4% and 57.0%, respectively (see Tables 1, 2).

**Table 1:** Comparison of demographic characteristics and poisoning-related factors between the two groups [*n* (%)].

Characteristic	Total (*n* = 726)	Non-severe group (*n* = 647)	Severe group (*n* = 79)	*χ*^2^ value	*P* value
Sex				0.124	0.724
Male	354 (48.8)	314 (48.5)	40 (50.6)		
Female	372 (51.2)	333 (51.5)	39 (49.4)		
Residence				3.137	0.077
Rural	590 (81.3)	520 (80.4)	70 (88.6)		
Urban	136 (18.7)	127 (19.6)	9 (11.4)		
Age group				10.451	0.015
Infants and toddlers	227 (31.3)	211 (32.6)	16 (20.3)^b^		
Preschool age	149 (20.5)	137 (21.2)	12 (15.2)^ab^		
School age	74 (10.2)	61 (9.4)	13 (16.5)^a^		
Adolescence	276 (38.0)	238 (36.8)	38 (48.1)^ab^		
Poisoning method				7.172	0.007
Self-administration	251 (34.6)	213 (32.9)	38 (48.1)		
Accidental	475 (65.4)	434 (67.1)	41 (51.9)		
Poison type				6.088	0.107
Pesticides	259 (35.7)	57 (8.8)	33 (41.8)*		
Other Drugs	212 (29.2)	68 (10.5)	15 (19.0)		
Antidepressants	116 (16.0)	24 (3.7)	11 (13.9)		
Others	139 (19.1)	77 (11.9)	20 (25.3)		
Left-behind children				15.571	<0.001
Yes	62 (8.5)	46 (7.1)	16 (20.3)		
No	664 (91.5)	601 (92.9)	63 (79.7)		
Exposure-to-admission time				2.940	0.401
≤1 h	98 (13.5)	91 (14.1)	7 (8.9)		
1–4 h	257 (35.4)	224 (34.6)	33 (41.8)		
4–6 h	200 (27.5)	181 (28.0)	19 (24.1)		
>6 h	171 (23.6)	151 (23.3)	20 (25.3)		

Data are presented as *n* (%). Pesticides included organophosphates, rodenticides, herbicides, and insecticides; Other drugs included sedatives, antibiotics, and antihypertensives; Others included chemical agents, animal bites, carbon monoxide, alcohol, and poisonous mushrooms. Different superscript letters (a, b) indicate significant pairwise differences after Bonferroni correction (*P* < 0.05). *P* < 0.05 in bold. * In severe group, organophosphates accounted for 28 (35.4%) of all pesticide poisoning cases.

**Table 2:** Comparison of clinical characteristics and treatment measures between the two groups [*n* (%)].

Characteristic	Non-severe group (*n* = 647)	Severe group (*n* = 79)	*χ*^2^ value	*P* value
Neurological involvement			138.026	<0.001
Yes	187 (28.9)	76 (96.2)		
No	460 (71.1)	3 (3.8)		
Respiratory involvement			105.263	<0.001
Yes	18 (2.8)	25 (31.6)		
No	629 (97.2)	54 (68.4)		
Cardiovascular involvement			74.211	<0.001
Yes	2 (0.3)	11 (13.9)		
No	645 (99.7)	68 (86.1)		
Gastrointestinal involvement			0.216	0.642
Yes	442 (68.3)	56 (70.9)		
No	205 (31.7)	23 (29.1)		
Skin or mucosal involvement			2.333	0.127
Yes	56 (8.7)	11 (13.9)		
No	591 (91.3)	68 (86.1)		
Gastric lavage			4.113	0.043
Yes	611 (94.4)	70 (88.6)		
No	36 (5.6)	9 (11.4)		
Antidote use			29.068	<0.001
Yes	177 (27.4)	45 (57.0)		
No	470 (72.6)	34 (43.0)		
Ventilator use			74.634	<0.001
Yes	0 (0.0)	9 (11.4)		
No	647(100.0)	70(88.6)		

Data in parentheses are percentages within the group. Between-group comparisons were performed using the chi-square test. A *P* value < 0.05 was considered statistically significant.

### 3.2 Univariate analysis of between-group differences

There were significant differences between the severe and non-severe groups in age group (*χ*^2^ = 10.451, *P* = 0.015), left-behind children (*χ*^2^ = 15.571, *P* < 0.001), poisoning method (*χ*^2^ = 7.172, *P* = 0.007), neurological involvement (*χ*^2^ = 138.026, *P* < 0.001), respiratory involvement (*χ*^2^ = 105.263, *P* < 0.001), cardiovascular involvement (*χ*^2^ = 74.211, *P* < 0.001), gastric lavage (*χ*^2^ = 4.113, *P* = 0.043), antidote use *(χ*^2^ = 29.068, *P* < 0.001), and ventilator use (*χ*^2^ = 74.634, *P* < 0.001). All *P* values were <0.05, indicating statistical significance.

No significant differences were observed between the two groups in sex, residence, exposure-to-admission time, poison type, gastrointestinal involvement, skin or mucosal involvement (all *P* > 0.05). See Tables 1, 2.

### 3.3 Risk factors for severe poisoning

With severity as the dependent variable (1 = severe, 0 = non-severe), neurological, respiratory, and cardiovascular involvement were not included in the regression model due to their collinearity with the PSS; ventilator use was also excluded due to its collinearity with respiratory involvement. Variables with statistical significance, including age group, poisoning method, left-behind children, gastric lavage, and antidote use, were entered into the binary logistic regression model (forward stepwise method). The results were as follows:

Gastric lavage was significantly associated with lower odds of severe poisoning (*OR* = 0.355, 95% *CI*: 0.157–0.800, *P* = 0.013); antidote use was associated with an increased risk of severe poisoning (OR = 3.156, 95% *CI*: 1.933–5.152, *P* < 0.001), while left-behind children (*OR* = 3.013, 95% *CI*: 1.558–5.828, *P* = 0.001) and self-poisoning (*OR* = 2.113, 95% *CI*: 1.283–3.481, *P* = 0.003) were independent risk factors. The model showed an area under the ROC curve of 0.710 (95% *CI*: 0.645–0.775). See Table 3. Collinearity diagnostics showed that the variance inflation factor (VIF) was <5 for all variables (Table 4), indicating no multicollinearity.

**Table 3:** Binary logistic regression analysis of independent risk factors for severe poisoning in children.

Variable	*β* value	Standard error	Wald value	*P* value	*OR* value	95% CI
Constant	−2.075	0.407	25.937	＜0.001	0.126	-
Gastric lavage (yes vs. no)	−1.037	0.415	6.232	0.013	0.355	0.157–0.800
Antidote use (yes vs. no)	1.149	0.250	21.133	<0.001	3.156	1.933–5.152
Left-behind children (yes vs. no)	1.103	0.337	10.737	0.001	3.013	1.558–5.828
Self-poisoning (vs. accidental ingestion)	0.748	0.255	8.632	0.003	2.113	1.283–3.481

Dependent variable: severe poisoning (1 = yes, 0 = no). Variable selection: Forward:LR.

**Table 4:** Collinearity diagnostics for variables with *P* < 0.05.

**Variable**	**Tolerance**	Variance inflation factor (VIF)
Gastric lavage	0.988	1.012
Antidote use	0.978	1.023
Left-behind children	0.977	1.023
Self-poisoning	0.985	1.015

The VIF for all variables was <5, indicating no serious multicollinearity among the independent variables.

### 3.4 Evaluation of the predictive performance of risk factors for poisoning

**Figure 1:**
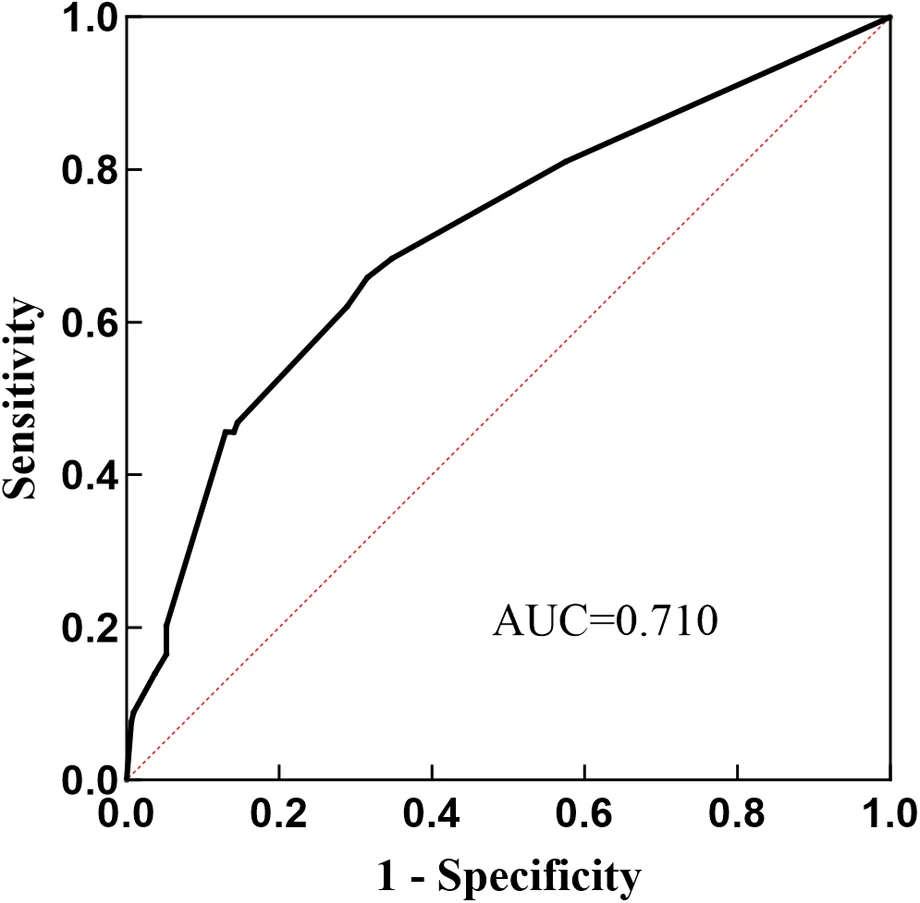
ROC curve of risk factors for severe acute poisoning.

The Hosmer-Lemeshow test showed *χ*^2^ = 5.646, *P* = 0.687(Table 5), indicating good model fit. The area under the ROC curve (AUC) was 0.710 (95% *CI*: 0.645–0.775, *P* < 0.001) (Figure 1), suggesting acceptable discriminative ability.

**Table 5:** Model goodness-of-fit and predictive performance.

**Evaluation index**	**Result**	**95% CI**	***P value***
**Hosmer-Lemeshow test**	*χ*^2^ = 2.198	—	0.699
**Area under the ROC curve (AUC)**	0.710	0.645–0.775	＜0.001

The Hosmer-Lemeshow test *P* > 0.05 indicates good model fit; AUC > 0.710 indicates acceptable discriminative ability.

## 4 Discussion

In this retrospective study of 726 children with acute poisoning, we found that gastric lavage was significantly associated with a lower risk of severe poisoning (*OR* = 0.355, 95% *CI*: 0.157–0.800, *P* = 0.013). Although this association raises the possibility of a protective effect, it must be interpreted cautiously given the observational study design. This finding is broadly in line the Chinese Expert Consensus on Diagnosis and Treatment of Acute Poisoning, which recommends early gastric lavage for certain poisonings, particularly pesticides such as organophosphates and paraquat (7). However, the consensus recommendations are largely derived from clinical experience, and our observational findings, while supportive of this practice, cannot be considered as direct causal evidence. Conversely, some international studies have suggested that gastric lavage may not always be beneficial and may be associated with complications (11). This discrepancy may be attributed to the fact that the poisoning spectrum in China is still dominated by pesticides, and the consensus recommends aggressive gastric lavage for pesticide poisoning, such as organophosphates and paraquat. In addition, due to the vast territory of China, most primary healthcare facilities lack advanced life support modalities such as blood purification, making gastric lavage the most direct and accessible method of poison elimination.

Therefore, these findings are consistent with the expert consensus recommendations on strictly adhering to the indications for gastric lavage (7, 12), and also remind clinicians that when performing gastric lavage in children with acute poisoning, comprehensive evaluation should be based on the type of poison, severity of illness, and contraindications. With strict adherence to indications, gastric lavage may still benefit some patients.

Contrary to clinical intuition, this study found that antidote use (*OR* = 3.156) was associated with an increased risk of severe poisoning. This result should be interpreted with caution: it does not mean that antidote use increases the risk of severe poisoning, but rather reflects a classic “confounding by indication” bias—severely ill patients are more likely to receive antidotes due to the severity of their condition. Geith et al. (10) reported that antidote use increased progressively with Poisoning Severity Score (PSS) grading: 9.8% in the asymptomatic group, 9.1% in the mild group, 27.7% in the moderate group, 44.3% in the severe group, and 57.1% in the fatal group. In our cohort, 28 cases (35.4%) of severe poisoning were due to organophosphate poisoning, and due to the lack of blood purification technology in our region, clinical management frequently required repeated administration of antidotes such as atropine and pralidoxime (7). The high rate of antidote use essentially reflects the severity of the disease rather than being treatment-induced. However, this does not mean that antidotes are ineffective. El-Sarnagawy et al. (13) demonstrated in a retrospective study of 447 poisoned patients that antidotes including atropine, oximes, and N-acetylcysteine significantly improved clinical outcomes. This suggests that clinicians, especially those in primary care settings with medical capabilities comparable to our institution, should administer antidotes early and appropriately based on the severity of the patient's condition (7).

In this study, self-poisoning (*OR* = 2.113) was identified as an independent risk factor for severe poisoning, indicating that self-poisoning increases the risk of severe outcomes, which is consistent with many domestic and international studies (14–17). In our cohort, adolescents accounted for a high proportion of the severe group. Adolescents are in a critical stage of psychological development, and psychiatric disorders such as depression and anxiety, as well as psychosocial factors including academic pressure and family conflicts, are important drivers of self-poisoning behavior (18). Self-poisoning is often associated with self-harm intent, with the ingested dose far exceeding therapeutic levels, and these patients frequently ingest multiple substances. The severity of poisoning is closely related to the dose of the toxicant(s), generally following a dose-response relationship (7). Current evidence indicates that polysubstance ingestion is one of the strongest independent predictors of severe outcomes in pediatric poisoning (19). In addition, our region is agricultural, with high accessibility to toxic substances; self-poisoning patients may delay seeking medical care due to shame or fear, and it has been reported that exposure time ≥8 h is an independent risk factor for life-threatening outcomes (17).

Clinicians should prioritize the care of children with self-poisoning by integrating mental health assessments and early psychological interventions alongside clinical treatment. Furthermore, psychological interventions should be combined with poisoning prevention efforts and incorporated into school- and community-based health education programs to reduce the incidence and recurrence of self-poisoning.

This study found that left-behind children were a risk factor for severe acute poisoning, which is consistent with the findings of Huang et al (20). The reasons may be related to the fact that our region is an economically underdeveloped agricultural area with a high proportion of migrant workers, leaving many children under the care of their grandparents. Grandparental caregivers are often older and have lower levels of education. They tend to have limited awareness of the hazards of toxic substances and often store poisons improperly (21, 22). In addition, caregivers lack pre-hospital first aid knowledge, leading to delayed recognition and delayed medical consultation after poisoning, which prolongs exposure time and further exacerbates the severity of the condition.

Poisoning prevention strategies should focus on left-behind children. First, strengthen caregiver safety education to improve their awareness of poison hazards, recognition of poisoning, and first aid skills. Second, enhance household poison management by instructing caregivers to store pesticides, medications, and corrosive substances out of children's reach. Finally, it is recommended that relevant authorities strengthen the regulation of pesticide sales and use, promote child-safe packaging, and reduce the excessive storage of highly toxic pesticides in households. Li et al. (22) also recommended that governments and society strengthen regulatory oversight to reduce the unregulated circulation of pesticides and drugs, and reinforce the dissemination of child safety knowledge.

Tseng et al. (17) based on 17,274 cases from Taiwan Province of China, further confirmed that organophosphates are among the most common toxicants associated with severe-to-fatal outcomes, and pesticide use is a significant risk factor for life-threatening outcomes (*P* < 0.01). However, in the present study, poison type was not significantly associated with severe poisoning (*P* = 0.107), which may be attributed to the relatively small sample size of the severe group (only 79 cases), and the further reduction in case numbers within each toxicant subgroup after classification, leading to insufficient statistical power. In our clinical data, pesticides accounted for the highest proportion of severe cases (41.8%, 33/79), among which organophosphates accounted for 84.8% (28/33) of pesticide-related severe cases. The high proportion of organophosphates is mainly attributable to the fact that our region is an agricultural area with high accessibility to organophosphates. Although the association between poison type and severity did not reach statistical significance, clinicians should still pay close attention to the high prevalence of organophosphates in this region and strengthen preventive management and health education.

The logistic regression model established in this study showed good fit according to the Hosmer-Lemeshow test (*P* = 0.699), and the area under the ROC curve (AUC) was 0.710 (95% *CI*: 0.645–0.775), indicating that the model has acceptable discriminative ability to distinguish between severe and non-severe poisoning cases. This finding suggests that clinicians, especially those in primary care settings in this region and other similar healthcare conditions, may use this model to identify high-risk factors, with particular attention to modifiable factors such as gastric lavage, left-behind children, and self-poisoning, to enable early and timely intervention.

This study has several limitations. First, it was a single-center retrospective study, so the generalizability of the results is limited. Second, laboratory indicators such as blood drug concentrations and blood gas analysis were not included, which may affect the assessment of the degree of organ dysfunction. Third, although this study clarified the timing of PSS assessments in the Methods, using the highest PSS within 24 h to define severe cases may introduce bias. For instance, a patient who improved from PSS 4 to 1 after gastric lavage and antidote treatment and another who worsened from PSS 1 to 4 at 24 h would both be classified as severe, yet their clinical trajectories are opposite. This may confound the estimated association between interventions (e.g., gastric lavage, antidotes) and poisoning severity. Ideally, the outcome should have been defined using PSS measured after these interventions; however, due to the retrospective design, this study could not standardize the timing of post-intervention PSS reassessment, and the 24-hour PSS was also influenced by other concurrent treatments, making it difficult to isolate the effect of gastric lavage or antidotes alone. Therefore, the finding—“gastric lavage is associated with lower odds of severe poisoning"—represents a statistical association and should not be interpreted as causal protection. Future prospective studies with standardized post-intervention PSS timing are needed for more definitive conclusions. Fourth, precise dose information (e.g., milligram amount or number of pills) was not collected, which may have affected the assessment of the dose response relationship. Fifth, psychosocial factors (e.g., depression scales, suicide risk assessments) were not included, limiting the analysis of self-poisoning behavior. Sixth, The sample size of the severe group was relatively small (only 79 cases), and further validation with larger, multicenter cohorts is needed.

## Data Availability

The raw data supporting the conclusions of this article will be made available by the authors, without undue reservation.
